# Identifying quality indicators to measure workplace violence in healthcare settings: a rapid review

**DOI:** 10.1186/s12873-024-00943-w

**Published:** 2024-02-16

**Authors:** Brendan Lyver, Jaswanth Gorla, Christian Schulz-Quach, Melanie Anderson, Brendan Singh, Trevor Hanagan, Jennifer Haines, Rickinder Sethi

**Affiliations:** 1 Department of Psychiatry, Faculty of Medicine, University of Toronto, Toronto, ON Canada; 2 Department of Supportive Care, Princess Margaret Cancer Centre, University Health Network, Toronto, ON Canada; 3 Centre of Mental Health, University Health Network, Toronto, ON Canada; 4 Temerty Faculty of Medicine, University of Toronto, Toronto, ON Canada; 5 University Health Network, 200 Elizabeth St, M5G 2C4 Toronto, ON Canada

**Keywords:** Workplace Violence in Health Care, Pandemic recovery, Quality Improvement, Quality indicators, Emergency Department

## Abstract

**Background:**

Workplace violence (WPV) in healthcare is a growing challenge posing significant risks to patient care and employee well-being. Existing metrics to measure WPV in healthcare settings often fail to provide decision-makers with an adequate reflection of WPV due to the complexity of the issue. This increases the difficulty for decision-makers to evaluate WPV in healthcare settings and implement interventions that can produce sustained improvements.

**Objective:**

This study aims to identify and compile a list of quality indicators that have previously been utilized to measure WPV in healthcare settings. The identified quality indicators serve as tools, providing leadership with the necessary information on the state of WPV within their organization or the impact of WPV prevention interventions. This information provides leadership with a foundation for planning and decision making related to addressing WPV.

**Methods:**

Ovid databases were used to identify articles relevant to violence in healthcare settings, from which 43 publications were included for data extraction. Data extraction produced a total of 229 quality indicators that were sorted into three indicator categories using the Donabedian model: structure, process, and outcome.

**Results:**

A majority of the articles (93%) contained at least 1 quality indicator that possessed the potential to be operationalized at an organizational level. In addition, several articles (40%) contained valuable questionnaires or survey instruments for measuring WPV. In total, the rapid review process identified 84 structural quality indicators, 121 process quality indicators, 24 outcome quality indicators, 57 survey-type questions and 17 survey instruments.

**Conclusions:**

This study provides a foundation for healthcare organizations to address WPV through systematic approaches informed by quality indicators. The utilization of indicators showed promise for characterizing WPV and measuring the efficacy of interventions. Caution must be exercised to ensure indicators are not discriminatory and are suited to specific organizational needs. While the findings of this review are promising, further investigation is needed to rigorously evaluate existing literature to expand the list of quality indicators for WPV.

**Supplementary Information:**

The online version contains supplementary material available at 10.1186/s12873-024-00943-w.

## Introduction

### Rationale

Workplace Violence (WPV) is a risk disproportionately affecting the healthcare sector that has been linked to poorer quality of patient care provision and affects the well-being of employees. The frequency and severity of violence in healthcare has been consistently increasing over the past decade [1]. Alarmingly, more recent data highlight even larger leaps in aggression and WPV across many clinical settings during the COVID-19 pandemic [2–4]. Emergency departments have been disproportionately impacted, with up to two-fold increases in violent incidents compared to pre-pandemic levels [3, 5]. Higher frequencies of violent incidents have continually been linked to reduced quality of patient care and decreased quality of life for healthcare employees [2]. Despite the prevalence and impact of WPV, studies have found that a significant number of organizational interventions fail to produce sustained improvements [6, 7]. Nonetheless, healthcare organizations continue to develop interventions that attempt to mitigate the effects of WPV on staff and patient safety, with varying efficacy that can be attributed to their attentiveness to these gaps.

**Fig. 1:**
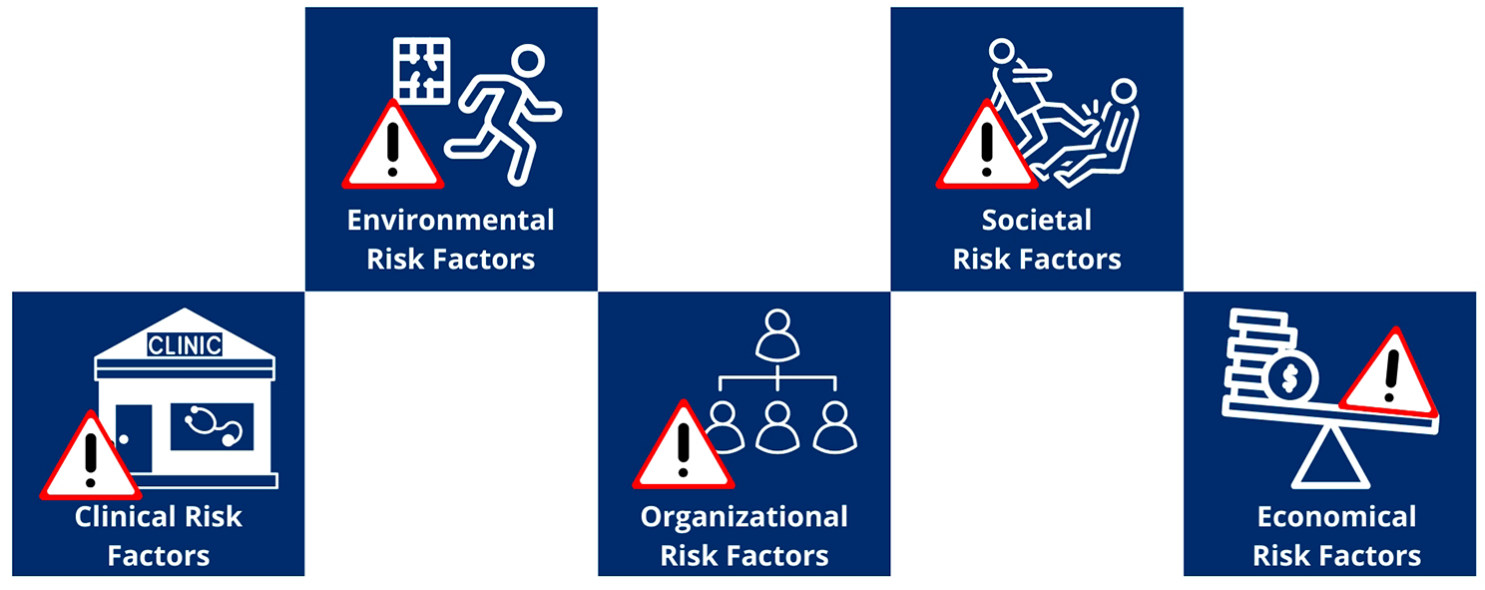
There exist 5 risk factors surrounding workplace violence in healthcare settings, this includes Clinical Risk Factors, Environmental Risk Factors, Organizational Risk Factors, Societal Risk Factors, and Economical Risk Factors

The complexity of WPV is frequently underestimated by healthcare organizations, with a tendency to attribute incident trends to single-factor causes. Consequentially, interventions that aim to address WPV tend to focus on individual-level responses such as updating staff education or modifying security presence. These interventions can serve as valuable components of a systemic response; but when implemented in isolation, do not create sustained improvements in outcomes as they fail to address the full range of factors behind WPV [8]. Literature suggests that risk factors for WPV can be broadly placed into five categories: (1) Clinical Risk Factors, (2) Environmental Risk Factors, (3) Organizational Risk Factors, (4) Societal Risk Factors, and (5) Economical Risk Factors [9]. Clinical risk factors include perpetrators being under influence of alcohol or drugs, severe pain, history of violence, cognitive impairment and certain psychiatric diagnoses, as well as lack of beds, improper placement of patients and long wait times. Environmental risk factors involve the physical workplace including variables such as insufficient heating or cooling, irritating noise levels, unsecured items, such as furniture, that may serve as a weapon of opportunity, or lack of security alarms. Organizational factors involve institutional guidelines, protocols and culture aspects such as number of staff with de-escalation training, effectiveness of policies in place regarding WPV, inappropriate placement of staff and staff working alone with agitated patients. Societal risk factors are related to the patients and visitors that arrive in the clinic such as the presence of weapons, patients or visitors possessing negative societal attitudes towards HCP or visible minorities working in healthcare, or police authorities using healthcare institutions as a temporary holding place for violent individuals. Economical risk factors include the lack of funding available for WPV prevention interventions, lack of staffing, lack of specialized staff and lack of resources such as physical restraints or seclusion rooms [9]. The 5 risk factors are summarized in Fig. 1. Investigating these categories using a systematic approach will enable organizations to contextualize the challenges of complex WPV interventions.

The Donabedian model has frequently been applied to understand the structures, processes, and outcomes in complex work systems in a healthcare setting [10]. The model serves as a framework to organize quality indicators based on the systems they impact.

Evaluating the efficacy of complex interventions addressing WPV is a complicated and multi-dimensional process frequently underdeveloped in healthcare organizations. Evaluation metrics for WPV are often limited to quantitative measures of incident rates or formal emergency responses. For instance, recommendations from workplace safety commissions regarding evaluating WPV are often limited to these surface-level metrics, including Ontario’s Public Services Health & Safety Association (PSHSA) which recommends monitoring WPV frequency using four measures: (1) flagged patient involvement, (2) usage of force, (3) root cause analysis utilization, and (4) code white utilization [11]. However, these metrics alone can misrepresent the efficacy of interventions, in part due to the unreliability caused by underreporting of violence against healthcare workers, particularly during the pandemic [12, 13]. Additionally, there may be paradoxically higher rates of WPV reported following successful interventions due to improvements to incident detection, reporting systems, modernized characterization of WPV, and fostering a reporting culture. A more comprehensive set of evaluation metrics, or quality indicators, should be utilized to measure WPV and guide decision-making. While work has been done to develop quality indicators for WPV interventions, there is a need for a scoping review of the literature to distill and compile findings that are compatible with a modern healthcare approach to patient safety.

### Objectives

Developing quality indicators for patient safety and WPV outcomes is challenging due to the multi-dimensional complexity of the interventions involved and the scope of the problem. For the purpose of this review, WPV is defined as an act or attempt of physical or verbal abuse or the use of threatening behaviour by a patient, caregiver, chosen family member or visitor towards a worker in their workplace. Within the five categories of WPV risk factors, there exists the potential for a plethora of initiatives to improve outcomes. Identifying quality indicators to evaluate these initiatives and their role within a complex intervention is important to their success [14], yet there are limited comprehensive resources identifying quality indicators for WPV interventions. Recent studies on WPV intervention efficacy note the sparsity of evidence regarding measurements of outcomes, which were instead supplemented with self-reported outcomes by participants [15]. Systematic literature review findings regarding WPV against healthcare professionals overwhelmingly rely on subjective measures, such as questionnaires and narrative evidence [16]. Therefore, this review aims to identify and compile a list of existing quality indicators that have been utilized to provide insight into WPV within healthcare organizations and the impact of interventions to address WPV.

## Methods

### Protocol and Registration

This rapid review was performed following the preferred reporting items for systematic review and meta-analyses extension for scoping reviews (PRISMA-ScR) guideline [17]. This review was approved as part of a quality improvement initiative by the UHN quality improvement review board (QI ID: 22–0499).

### Eligibility criteria

Studies eligible for review included examinations of workplace violence in healthcare through a quality improvement approach. The following study designs were eligible for inclusion in this review: abstracts, case studies, cluster-randomized control studies, commentaries, comparative studies, cross-sectional studies, database analysis, Delphi method studies, letters to the editor, literature reviews, meta-analysis, mixed method studies, qualitative studies, quality improvement studies, quantitative studies, quasi-experiments, systematic reviews, and validating method studies. Literature was selected for further review based on the inclusion of keywords relevant to workplace violence in healthcare in study titles and abstracts. Studies were excluded during screening if they did not utilize quality indicators, were not primary sources of evidence, or if the study did not report quality indicators. For the purpose of this review, a quality indicator was defined as “a quantitative measure that provides information about a variable that is difficult to measure directly” [18].

### Search methods

Precise searches were designed by an experienced health sciences librarian in Ovid databases, Medline, Embase, and Emcare based on two target papers and input from the research team. Subject headings and keywords were selected for concepts of psychiatric emergency or violence, hospital settings, and quality measures, with the aim of maximizing the specificity of the search results while also not focusing on any one kind of violence, setting or measure. Major heading was applied to all subject headings, and keywords were restricted to titles and assigned keywords. Results were limited to those written in the English language.

The challenges for this search included examples of violence being addressed in the body of the papers but not in the searchable fields of the database records, the frequency with which terms related to the concepts of interest appear in unrelated article records, and the speed with which the review stage of the Quality Improvement project needed completion. Search decisions were therefore aimed at high relevancy, and away from high sensitivity. Database records were exported on February 22, 2023, and loaded into Covidence for screening. All data extracted was contained within articles and there was no need to contact authors for missing information.

### Search

Database(s): Ovid MEDLINE(R) ALL 1946 to February 21, 2023.

Search Strategy:

**Table:** 

#	Searches	Results
1	exp *Emergency Services, Psychiatric/	1960
2	(psych* adj3 emergenc*).ti,kf.	2392
3	(agitat* or aggress* or violen* or abuse or abusive* or assault* or bully* or harass*).ti,kf.	145,194
4	*violence/ or exp *domestic violence/ or *gender-based violence/ or *gun violence/ or exp *intimate partner violence/ or *physical abuse/ or *rape/ or *workplace violence/	72,759
5	exp *Aggression/	26,686
6	or/1–5	179,579
7	exp *Hospital Departments/	126,362
8	(hospital or hospitals).ti,kf.	346,986
9	((inpatient or in-patient or medical or surg* or critical* or intensive*) adj2 (department* or unit* or ward* or floor*)).ti,kf.	53,844
10	(emerg* adj2 (department* or dept or unit* or room?)).ti,kf.	50,323
11	or/7–10	514,626
12	6 and 11	6751
13	exp *"Quality of Health Care”/	983,252
14	exp *Health Services Administration/ and quality.ti,kf.	74,010
15	(quality adj2 (measur* or indicat* or scale? or assess* or framework* or benchmark*)).ti,kf.	20,775
16	or/13–15	1,006,998
17	12 and 16	613
18	limit 17 to english language	581

Database(s): Embase Classic + Embase 1947 to 2023 February 21.

Search Strategy:

**Table:** 

#	Searches	Results
1	*emergency psychiatry/	92
2	exp *mental health care/ and emerg*.ti,kf.	1253
3	exp *violence/	96,307
4	exp *aggression/	43,047
5	(psych* adj3 emergenc*).ti,kf.	3273
6	(agitat* or aggress* or violen* or abuse or abusive* or assault* or bully* or harass*).ti,kf.	191,738
7	or/1–6	246,584
8	exp *hospital/	360,838
9	(hospital or hospitals).ti,kf.	454,827
10	((inpatient or in-patient or medical or surg* or critical* or intensive*) adj2 (department* or unit* or ward* or floor*)).ti,kf.	79,536
11	(emerg* adj2 (department* or dept or unit* or room?)).ti,kf.	73,320
12	or/8–11	740,773
13	exp *health care quality/	722,670
14	exp *"organization and management”/ and quality.ti,kf.	37,167
15	exp *quality control procedures/	583,996
16	(quality adj2 (measur* or indicat* or scale? or assess* or framework* or benchmark*)).ti,kf.	29,840
17	or/13–16	1,270,134
18	7 and 12 and 17	557
19	limit 18 to english language	517

Database(s): Ovid Emcare Nursing 1995 to Present.

Search Strategy:

**Table:** 

#	Searches	Results
1	*emergency psychiatry/	17
2	exp *mental health care/ and emerg*.ti,kf.	404
3	exp *violence/	50,924
4	exp *aggression/	15,893
5	(psych* adj3 emergenc*).ti,kf.	1060
6	(agitat* or aggress* or violen* or abuse or abusive* or assault* or bully* or harass*).ti,kf.	95,159
7	or/1–6	108,162
8	exp *hospital/	95,816
9	(hospital or hospitals).ti,kf.	126,115
10	((inpatient or in-patient or medical or surg* or critical* or intensive*) adj2 (department* or unit* or ward* or floor*)).ti,kf.	33,314
11	(emerg* adj2 (department* or dept or unit* or room?)).ti,kf.	34,114
12	or/8–11	213,062
13	exp *health care quality/	139,818
14	exp *"organization and management”/ and quality.ti,kf.	13,585
15	exp *quality control procedures/	109,085
16	(quality adj2 (measur* or indicat* or scale? or assess* or framework* or benchmark*)).ti,kf.	10,565
17	or/13–16	243,077
18	7 and 12 and 17	159
19	limit 18 to english language	143

### Selection of sources of evidence

Three reviewers independently screened all abstracts for articles relevant to workplace violence in healthcare settings. Articles that received two or more “include” votes were included for full-text review. To maintain consistency, all 63 articles that proceeded to a full-text screening were independently reviewed by the three reviewers to determine the inclusion of quality indicators. Articles that did not contain quality indicators were excluded. Disagreements on study selection were resolved through consensus following reviewer discussion. An additional article from the Ontario Public Health and Safety Association was added to the data extraction phase as well due to the valuable quality indicators that the document possessed. The final 43 articles underwent extraction of quality indicators by the three authors. Afterwards, the authors organized quality indicators into one of three categories: structure quality indicators, process quality indicators, or outcome quality indicators.

### Data charting process

Data charting was facilitated through the Covidence platform which allowed custom entry fields to be included in charts. Data from eligible studies was extracted and charted by three independent reviewers. The data charting tool allowed for the collection of standardized study data and quality indicators that were identified by reviewers. Charted data was continuously and iteratively updated as each reviewer completed their screening of studies. Any disagreements were discussed amongst the reviewers to arrive at a consensus that was updated in the chart.

### Data items

The following information was extracted: article type, country of study, study design, study population, structural quality indicators, process quality indicators, and outcome quality indicators. This study was a rapid review, so the risk of bias was not assessed.

### Synthesis of results

Reviewers investigated each study that was included for a full-text review to extract quality indicators that were either explicitly reported or substantially implied based on study context and application. Data charts for each study were populated with extracted these quality indicators. After reviewing all included studies, reviewers categorized indicators into three categories based on the Donabedian model for quality of care: structural, process, or outcome. The reviewers extracted survey instruments or questions as well that were grouped separately from the quality indicators. Both categorizations were based on majority consensus between the three reviewers. The classification of quality indicators into the three delineated categories as per the Donabedian model necessitated thorough discussions among the reviewers. Although consensus was ultimately achieved for each individual indicator, the reviewers were aware that certain indicators may be susceptible to alternative categorizations owing to inherent ambivalence. The final quality indicator list and the list of survey instruments and questions were presented in separate table formats.

## Results

### Selection of sources of evidence

**Fig. 2:**
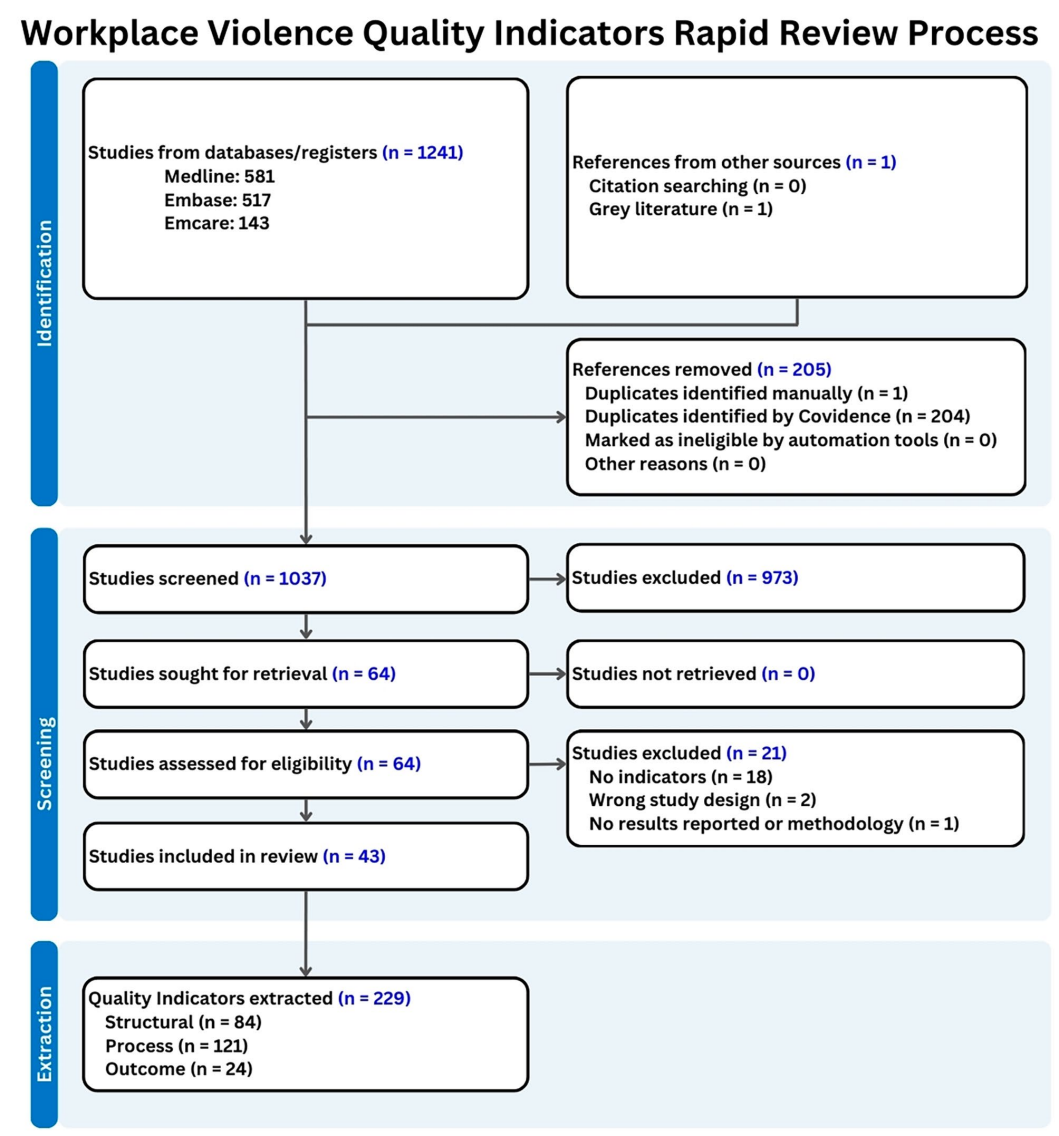
Study search and screening process completed with Covidence

In total, 1241 abstracts were identified for consideration in addition to one article from grey literature. 205 duplicates were removed from this pool, leaving 1037 papers for title and abstract screening. From this, 973 abstracts were deemed irrelevant, and 64 proceeded to a full-text review. Of the 64 studies, 21 were excluded for ineligibility, 18 of which had no indicators, 2 of which had the wrong study design, and 1 which did not report results or methodology. The final 43 studies were reviewed for data extraction and analysis. This process is summarized in Fig. 2.

### Characteristics of sources of evidence

The characteristics of the articles that were reviewed for data extraction including the article type, country of study, study design, and study population are described in Table S1 and Table S2.

### Results of individual sources of evidence

Table S1 includes the quality indicators extracted from the studies organized into one of three categories; Structural Quality indicators, Process Quality Indicators, and Outcome Quality Indicators (Table S1).

TABLE S1 PLACEHOLDER PLEASE REFER TO EXCEL “Supplementary File - Table S1.xls”.

While some papers did not provide quality indicators, they provided validated questionnaires or references to validated questionnaires that can be useful when investigating healthcare providers, staff, students, learners, and volunteers’ perceptions related to workplace violence. Please see Table S2 for more information.

TABLE S2 PLACEHOLDER PLEASE REFER TO EXCEL “Supplementary File - Table S2.xls”.

### Synthesis of results

The aggregation of results captured a wide scope of quality indicators for workplace violence (WPV) interventions consistent with the three major components of the Donabedian model [10]. The 43 studies identified for data extraction produced 229 raw quality indicators. Delineation of these indicators into operationalizable categories has the potential to assist in developing practical approaches to WPV. The 229 quality indicators were categorized using the Donabedian model, which outlines three major components of human-centred systems: Structural (84 indicators), Process (121 indicators), and Outcomes (24 indicators). It was crucial to set criteria for each category to assist with categorization. Structural indicators involved static and technical aspects of care provision, including attributes of staffing or the healthcare institution. Process indicators involved the steps taken when caring for a patient during, such as actions taken during aggressive incidents. Outcome indicators involved the impact of care on the patient, worker, and population, such as injury and post-incident support. A thematic analysis of each Donabedian component was completed to operationalize the review results further to produce clusters of indicators shown in Table S1. Categorizing and clustering indicators can help healthcare systems, target areas that are under-supported, underperforming, and in need of intervention. This use of specific indicators allows for deeper understanding and resource allocation efficient compared to general indicators that may have less utility, broader resource focus, and lack fulsome understanding.

The 84 indicators related to structural components primarily focused on evaluating objective and subjective measures of organizational preparedness for WPV incidents. Given the people-centred nature of incidents, staff education and perception significantly contributed to WPV readiness. Completion rates of risk-profile specific WPV training, including refresher training, were common indicators, along with staff evaluation of training quality and confidence. Frequently, indicators were used to gauge staff perception of WPV policies, guidelines, resources, and protocols. Due to the difficulty of collecting frequent qualitative insights from staff, these indicators qualitatively measure the percentage of staff describing satisfaction with interventions at 70% or more. Non-person structural indicators primarily involved evaluating the efficiency of patient flow through departments with a high reported number of WPV incidents, namely the ED and psychiatric units. It has been found that inefficiencies in patient flow have been observed to contribute to escalation of aggression and WPV [7]. It is worth noting that indicators such as patient flow rates or wait times measure Finally, it was important to include indicators that gauge the utilization of interventions implemented in response to WPV challenges.

The 121 indicators related to process components involved quantifying the various characteristics of WPV incidents and the utilization of interventions at different risk levels. Characteristics of WPV incidents were quantified by measuring frequencies of specific occurrences. These included the types of violence observed, such as different forms of verbal and physical assault, the targets of violence, and indications for security intervention, such as restraint or discharge assistance. Indicators for the utilization of WPV interventions were segregated into two risk levels: (1) lower risk events that did not escalate to formal responses such as Code Whites and (2) higher risk events that involved formal responses. For both categories, indicators aimed to quantify the frequency and timeliness of specific intervention utilization, such as de-escalation, seclusion, restraint, and medication. For health systems without tiered WPV response levels, intervention utilization indicators are applicable for all risk levels. A small set of indicators were also utilized to gauge healthcare worker perception of security team responses during violent incidents.

The 24 outcome indicators focused on adverse events affecting stakeholders involved in WPV incidents. Adverse events were categorized by the type of harm, physical or psychological, and the stakeholder group impacted, such as patients, staff, or other individuals present. Indicators were also selected to reflect adverse events caused by interventions specifically. Other outcome indicators involved quantifying the frequency of post-incident intervention utilization such as staff supports, debriefs, and formal reports. A selection of indicators was also dedicated to quantifying staff satisfaction with post-incident outcomes and responses.

The data extraction also led to the discovery of 57 survey questions and 17 survey instruments used to collect feedback from healthcare providers, staff, patients or their (chosen) family. The questions and instruments for healthcare providers and staff inquired about feelings of safety and anxiety regarding WPV, satisfaction with their organization, exposure to workplace violence, confidence managing aggressive behaviours, and inquired about staff’s perspectives on effective methods to address WPV. Survey questions for patients and (chosen) family members focused on their satisfaction with the care provided, and aggressive tendencies.

## Discussion

### Summary of evidence

This review compiled a comprehensive and actionable set of quality indicators with the potential to collect data at the structural, process, and outcome levels. These categories were adapted from the Donabedian model, which provides a reliable framework to understand patient safety from a technical approach, facilitating evaluation, planning, and research. 229 indicators were sorted into three categories: 84 being structural-related, 121 being process-related, and 24 being outcome-related. Process indicators tended to be more granular in their measurements, for instance, specific violent acts and interventions had their own unique indicators, which were further divided by risk levels. By comparison, structural and outcome indicators were less frequently utilized and tended to be less specific, suggesting these categories may be underutilized by contemporary approaches to WPV. Studies that reported structural indicators mainly derived utility from staff perceptions of WPV training and policies, while studies reporting outcome indicators utilized measures of adverse events, including lapses of safety and lost time. This review establishes a compilation of indicators across all three categories to serve as a starting point for health systems looking to incorporate comprehensive and actionable quality indicators.

Reviewers also compiled a list of validated survey instruments and questions from the literature. Some information cannot be captured through quantitative metrics; thus, it is important to collect qualitative data and feedback through methods such as surveys from healthcare providers, staff, volunteers, patients, caregivers and (chosen) family members to properly evaluate interventions and the current state of healthcare settings as they pertain to WPV. Articles included in this review utilized survey instruments and questions to measure the subjects’ feelings such as stress, safety or fear of violence, in addition to capturing subjects’ perspectives on the effectiveness or ease of use of certain interventions. An outcome of the growing prevalence of WPV in healthcare settings is that staff’s morale and feelings of safety have diminished [9]. While quality indicators can measure the impact of an intervention and trends in workplace violence, it is important to collect complimentary bottom-up data through routine surveys or qualitative interviews to capture a fulsome view of WPV in healthcare settings.

The quality indicators and validated survey instruments and questions extracted in this review will be valuable to healthcare institutions’ ability to adequately measure and evaluate WPV in their organizations. In recent decades, health systems have increasingly relied on data-driven systematic approaches to facilitate the continual improvement of their services. The steady increase in healthcare utilization [19] has made the quality of care and resource stewardship top priorities when providing efficient patient-centred care. Quantifying these complex and multi-dimensional metrics is a challenge that policymakers and investigators face when developing quality assurance and improvement strategies. Quality indicators have served as reliable metrics, allowing stakeholders to understand how effectively specific functions of health systems perform. Contemporary advancements in information technology and quality assurance theory have allowed indicators to become compelling and actionable sources of evidence. For instance, quality indicators have been pivotal to identifying gaps in acute care provision in emergency departments, enabling interventions to reduce wait times and improve triage across health systems [20]. Organizations can effectively use quality indicators to promote continuous efforts for stakeholders to improve performance and optimize outcomes [21]. Despite their well-documented potential, many health systems have yet to leverage quality indicators to tackle the increasingly prevalent issue of workplace violence in healthcare. This was apparent during our review of the relative sparsity of studies leveraging quality indicators within this domain. Furthermore, we noted a limited amount literature defining comprehensive and pertinent sets of indicators for measuring WPV in healthcare. Despite this, our review identified diverse and complex sets of indicators that were influential in measuring the burden of WPV in healthcare settings. These indicators were foundational to successful quality improvement (QI) initiatives within these settings. For instance, multiple studies reported indicators that measured the frequency of specific violent events and interventions. In one case, investigators utilized these indicators to demonstrate a significant reduction of restrictive interventions, patient self-harm, and staff injury after implementing patient-specific behaviour plans at a psychiatric hospital [22]. Other studies used indicators to measure changes in specific violent behaviours, such as bullying, verbal abuse, and physical abuse, in response to the implementation of risk assessment tools [23]. Another set of indicators focused on measuring staff perception of training on WPV prevention protocols and tools [24]. In several studies, training programs that received high staff approval were linked to increased usage of interventions and significant reductions in WPV incidents [23, 25]. Across all studies, quality indicators served to identify areas for improvement, track the quality of interventions, or contextualize resource allocation for specific challenges. Many studies applying these indicators reported positive outcomes with regards to reducing the burden of WPV and improving patient care outcomes.

It is crucial to be cognizant of psychosocial factors and to engage a modern healthcare lens when utilizing the quality indicators listed in this review. WPV incidents are stressful, acute situations where the impact of unconscious biases can result in unwanted outcomes. Quality indicators predicated on these biases can harmfully attribute likeliness of aggression to certain patient characteristics and validate interventions that target specific demographics. For example, an indicator measuring the incidence of WPV related to care of psychiatric patients may suggest interventions that target patients with mental health issues regardless of their actual risk. Such interventions can lead to stigma and patient mistreatment that exacerbates the health disparities faced by commonly marginalized groups [26]. Therefore, it was important to identify and exclude literature and indicators that were incompatible with modern care delivery standards to minimize damaging effects to patient psychosocial safety. In this context it is noteworthy that WPV research investigating actual incidence of violence in the United States in the last 20 years have unequivocally found that WPV is not related to race, ethnicity, or community (with only weak associations with community crime rates) [27]..

Three conceptual approaches to health disparity were given particular attention during the review process: (I) trauma-informed approach, (II) intersectional identities theory, and (III) minority stress theory. The trauma-informed approach recognizes that a patient’s individual circumstances can influence how they interact with healthcare services. Achieving this involves shifting away from blaming patients in favor of understanding the stressors underlying their behavior. The goal of the approach in the context of WPV is to use organizational policy and interventions to provide safe and effective care without re-traumatizing patients. This is of particular importance as re-traumatization has been shown to contribute to violent incidents when inappropriate treatment of patients living with trauma can trigger flight or fight responses [28]. Studies that focused on risk stratification based on staff perceptions of certain demographics were excluded from the review due to the potential of informing discriminatory and traumatizing interventions. For instance, one study asked hospital staff to record their agreement with the following statement: “patients from particular ethnic minority groups are more likely to become aggressive” [29]. Another study suggested the use of indicators and risk assessment based on behavioral cues including eye contact, tone and volume, anxiety, mumbling, and pacing [26]. In both cases, intrinsic patient characteristics were broadly framed as problematic, while their lived experiences and traumas were not adequately taken into consideration. The intersectional identities and minority stress theories both highlight the importance of acknowledging these unique experiences and stressors to understand how they influence health outcomes. In the context of WPV, they point to the historic mistreatment of certain identities and groups as stress-inducing factors that contribute to disproportionate rates of violence. Therefore, we posit that quality indicators should look to measure the effects of these root causes and their contribution to WPV incidents rather than focusing on the actions of specific demographics, which may contribute to further marginalization.

The introduction of novel quality indicators may require organizations to invest in solutions to manage the resulting higher volumes of data. Recent literature supports the utilization of automation and data visualization to systematically collect and report data from quality indicators in a way that is conducive to decision-making. A study conducted at a large hospital network found that automated data abstraction of quality measures significantly reduced processing time by up to 50% when compared with manual processing [30]. Research in the field of quality management suggests performance dashboards as effective instruments to visualize data in a way that is easily disseminated and digested by organizational decision-makers [31]. Despite its detailed coverage, it is important to note that in the emerging field of healthcare WPV QI, this reviews list of indicators is non-exhaustive and not broadly applicable to every healthcare environment. Quality indicators have varying utility based on the function of the healthcare system they serve [21]. For instance, indicators originally designed for acute or inpatient care settings, where security resources are more abundant, may fail to address the unique needs of less-equipped outpatient primary care. Therefore, organizations should rely on discussions with key stakeholders to distill and adapt quality indicators to fit their specific needs. Systematic approaches, such as the Delphi technique, can be leveraged to develop consensus amongst diverse stakeholders involved with WPV management. Ultimately, the set of indicators we identified through this review can serve as a foundation for healthcare organizations looking to manage WPV through a quality improvement approach.

### Limitations

This rapid review had several limitations. To make this review feasible, our methodology was expedited in several ways. The initial sourcing of articles was limited to three Ovid databases with search strings and inclusion criteria that may not capture the full breadth of existing literature. This may lead to findings not being fully generalizable to the existing state of literature. Furthermore, studies that were inaccessible publicly or through our institution could not be included in the review. Time constraints of this review meant that each study was reviewed expeditiously, and quality appraisal of included studies were limited, increasing the risk of bias and reduced confidence in findings. The time-sensitive manual extraction of quality indicators may lead to bias in findings and unintentional exclusion of pertinent indicators. Lastly, several indicators included in this review can be considered as indirect indicators of WPV. Given the expansive nature of WPV, the review team recognizes the potential value of these indicators while concurrently acknowledging that their direct correlation with workplace violence may not be unequivocal.

## Conclusions

The increasing prevalence of workplace violence over the past decade challenges healthcare systems worldwide. Quality improvement initiatives that aim to address and mitigate WPV will require a method of measuring WPV and the impact of interventions. This rapid review provides a resource for organizations looking for quality indicators previously used in literature to measure WPV. We reviewed existing literature to identify indicators for measuring structural, process, and outcome metrics and analyze their utility in care settings. We found evidence that indicators played important roles in planning and assessing interventions, often used to quantify specific improvements such as harm reduction, improved resource usage, and improved staff perceptions. Organizations looking to introduce WPV-related quality indicators should adapt and mold indicators to fit the unique needs of their care settings, ideally through systematic consultation with key stakeholders, including clinicians, quality researchers, and administrators. The quality indicators presented in this review have face validity with the potential to inform improvements to data collection relevant to WPV across various clinical care settings. Further investigations should rigorously review the full body of literature pertinent to WPV in healthcare to identify a more exhaustive list of quality indicators. These indicators can be leveraged to enhance the understanding of factors contributing to WPV and improve the applicability of indicators across more care environments. In addition, future studies should continue to assess the utility of structural, process, and outcome indicators in WPV quality improvement interventions.

### Electronic supplementary material

Below is the link to the electronic supplementary material.

Supplementary Material 1

Supplementary Material 2

## Data Availability

All data generated or analyzed during this study are included in this published article [and its supplementary information files].

## Abbreviations

WPV: Workplace violence
QI: Quality improvement
